# Central retinal vein occlusion in the setting of COVID-19 infection

**DOI:** 10.1186/s12348-021-00241-7

**Published:** 2021-04-02

**Authors:** Nilesh Raval, Anna Djougarian, James Lin

**Affiliations:** 1 Department of Ophthalmology, Montefiore Medical Center, Albert Einstein College of Medicine, 3332 Rochambeau Ave, 3rd Floor, Bronx, NY 10467 USA; 2 Department of Ophthalmology, Northwell Health, Great Neck, NY USA

To the Editor:

We report a case of central retinal vein occlusion (CRVO) in the setting of symptomatic coronavirus disease 2019 (COVID-19) infection in an otherwise healthy adult male.

## Case report

A 39-year-old male with no past medical or ocular history developed a fever in the Dominican Republic. The following day, the patient traveled back to the United States and tested positive for severe acute respiratory syndrome coronavirus 2 (SARS-CoV-2) by reverse transcriptase polymerase chain reaction (RT-PCR) assay. A week after the positive test, the patient developed decreased vision and floaters in the right eye and was referred for retinal exam 1 month later.

**Fig. 1:**
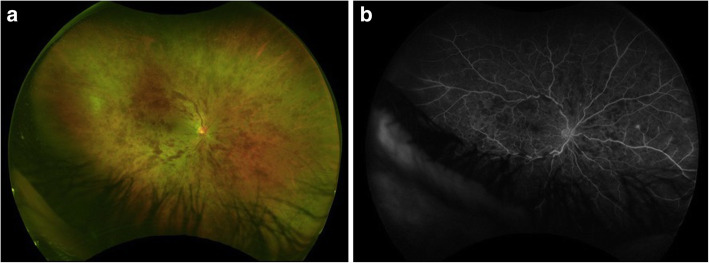
**a** Color fundus photograph OD showing a hyperemic optic nerve head, macular thickening, vascular tortuosity, and diffuse intraretinal hemorrhages. **b** Late-phase fluorescein angiogram OD demonstrating vascular tortuosity and vessel wall staining without capillary non-perfusion

**Fig. 2:**
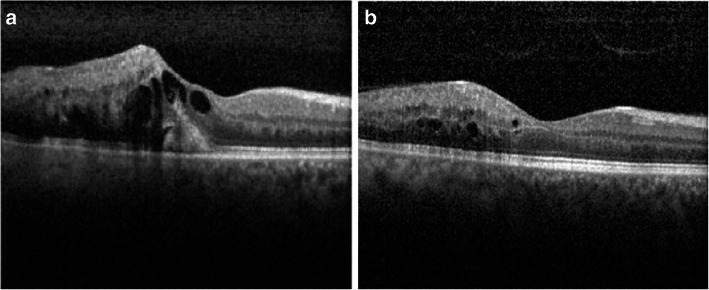
**a** Optical coherence tomography (OCT) OD showing CME on initial presentation. **b** OCT OD showing improvement of CME after intravitreal injection of bevacizumab

On initial presentation, visual acuity was 20/150 in the right eye (OD) and 20/30 in the left eye (OS). Pupils were equal, round, and reactive to light, and intraocular pressures were 16 mmHg OD and 17 mmHg OS. Slit lamp biomicroscopy of the anterior segment was normal except for nasal pterygia in both eyes. Dilated fundus examination OD revealed a hyperemic optic nerve head, macular thickening, tortuous vasculature, and diffusely scattered intraretinal hemorrhages (Fig. 1a). Fluorescein angiography OD showed tortuous vasculature and vessel wall staining without neovascularization, leakage, or areas of non-perfusion (Fig. 1b). Optical coherence tomography OD demonstrated cystoid macular edema (CME) (Fig. 2a). Fundus exam and ancillary testing OS were unremarkable.

Diagnostic workup, including blood pressure, complete blood count, glucose level, QuantiFERON Gold, anti-Treponemal IgG and IgM antibodies, antinuclear antibodies, anti-double stranded DNA antibodies, angiotensin converting enzyme, rheumatoid factor, homocysteine, C-reactive protein, erythrocyte sedimentation rate, and thrombophilia panel, were unremarkable. The patient was treated with intravitreal anti-vascular endothelial growth factor (anti-VEGF) injections of bevacizumab. After a series of injections, the macular edema decreased significantly and the patient’s visual acuity improved to 20/30 OD (Fig. 2b).

## Discussion

COVID-19-associated coagulopathy has been documented and studied since the onset of the pandemic [1]. Autopsy findings of patients with severe COVID-19 infection have demonstrated a hallmark diffuse small vessel thrombosis thought to be caused by complement-mediated microvascular injury, with platelet-fibrin microthrombi regularly found in venules, arterioles, and capillaries [2]. In addition, affinity of SARS-CoV-2 for vascular endothelial cell angiotensin-converting enzyme-2 (ACE2) receptors has been shown to activate apoptotic pathway signaling and prothrombotic cascades [3].

Hypercoagulability is a major risk factor for CRVO, and it is imperative to rule out other causes of CRVO in an otherwise healthy patient. Our patient did not have any significant medical history and infectious, inflammatory, and hypercoagulable studies were negative. There have only been a small number of cases of COVID-19-associated CRVO in recent literature [4–6]. Our patient’s positive RT-PCR SARS-CoV-2 assay and subsequent visual symptoms support the possibility that the pro-thrombotic state created by COVID-19 contributed to the development of CRVO.

This case builds upon the existing literature that demonstrates the occurrence of CRVO in the setting of COVID-19. Patients with COVID-19 are at risk for vascular occlusive events, and early detection and treatment are paramount in restoring visual function.

## Data Availability

Not applicable.

## Abbreviations

CRVO: Central retinal vein occlusion
COVID-19: Coronavirus disease 2019
SARS-CoV-2: Severe acute respiratory syndrome coronavirus 2
RT-PCR: Reverse transcriptase polymerase chain reaction
OD: Right eye
OS: Left eye
Anti-VEGF: Anti-vascular endothelial growth factor
ACE2: Angiotensin-converting enzyme-2
